# P-265. Assessment of the epidemiologic surveillance system for prevention of HIV transmission through blood transfusion, Almaty, 2024

**DOI:** 10.1093/ofid/ofaf695.486

**Published:** 2026-01-11

**Authors:** Dilnaz Aitbaeva, Ulyana Gubareva, S A N A M ZIKRIYAROVA, Roberta Horth, Alfiya Denebaeva, Dilyara Nabirova

**Affiliations:** Central Asia FETP, Almaty, Almaty, Kazakhstan; Central Asia FETP, Almaty, Almaty, Kazakhstan; Kazakh National Medical University named after S.D. Asfendiyarov;, Almaty, Almaty, Kazakhstan; US Centers for Disease Control and Prevention, Dulles, Virginia; Central Asia FETP, Almaty, Almaty, Kazakhstan; CDC Central Asia office, Almaty, Almaty, Kazakhstan

## Abstract

**Background:**

An estimated 33,658 people were living with HIV in Kazakhstan in 2024. Although HIV transmission through blood transfusion is rare, three cases of HIV were identified in hospitalized patients who received blood transfusions in 2023, raising concerns about blood transfusion as a potential source of transmission. Blood transfusion recipients are screened for HIV before transfusion and at 1- and 3-month post-transfusion. We aimed to identify weaknesses and strengths in Almaty, Kazakhstan’s surveillance system for HIV detection in blood transfusion recipients.

**Methods:**

In 2024, we conducted a descriptive cross-sectional survey to evaluate the surveillance system using updated guidelines from the US Centers for Disease Control and Prevention. Twenty key informants were interviewed from the city's blood and AIDS centers, two polyclinics, and a hospital.

**Results:**

The epidemiologic surveillance system for monitoring HIV transmission through the blood transfusion pathway is complex and multitiered. Patients receive transfusions in hospitals, and their test results are transmitted by phone to polyclinics for post-transfusion follow-up. The system utilizes separate medical and laboratory information databases that cannot be integrated due to patient protections, which makes patient follow-up difficult. Training for medical workers occurs 1-2 times a year, with 95% aware of the prevention algorithm. In 2023, of 71,234 blood recipients, 82 tested HIV positive in pre-transfusion testing (detection rate of 0.11%). Additionally, among 268,564 first-time blood donors, 54 tested HIV positive (detection rate of 0.02%). Gaps in data completeness were identified, with missing information in several variables and private medical facilities.

**Conclusion:**

The system effectively screens blood donors and recipients for HIV. Integrating medical and laboratory information systems while adhering to data protection protocols may help enhance coordination between medical facilities. Automated data transfer of HIV test results from laboratories and hospitals to patient polyclinic medical records might reduce processing time and minimize errors.

**Disclosures:**

All Authors: No reported disclosures

